# Clinical Cases

**Published:** 1882-11

**Authors:** George G. Gale

**Affiliations:** Quebec, Canada


### CLINICAL CASES.

George G. Gale, C. M., M. D., Quebec, Canada.

Case I.—Man, æt. fifty; has diplopia. Worse when turning the eyes to the right. *Causticum* 80m. Next day could see quite well.

Case II.—Woman, æt. thirty-five. Seven months enceinte. The abdomen is flattened in front, and bulging out in each lumbar region. The movements of the fœtus are very violent in the right lumbar region. Gave her one dose of Puls. 16m (Jenichen) at bed-time. Next day, no bulging of the lumbar regions; the abdomen has lost its flattened appearance, and the fœtus has ceased its violent movements. Continued well.

Case III.—Some time ago had an attack of sore throat. Two nights after my throat became sore I dreamed that a large snake was in the bed. The symptoms of my throat were as follows: The right tonsil red and swollen; pain in the tonsil of a gnawing character; worse at night. It tormented me so much that I could not sleep. For this condition of things I took Bell. It did me no good. I then tried Phytolacca, with the same result. It was now ten days I had been suffering. Thinking the case over, and recollecting the dream I had of the snake, made me try Lac. can. Cm (Swan). I took *one* dose at 10 P. M. At 11 P. M. the pain had almost disappeared. I went to bed and slept the whole night through, without pain, for the first time since ten days.

CASE IV.—A little girl has had running from the *left* ear, of a brown-colored, offensive discharge, for almost four years. Psorinum 45m cured her in a few weeks.

CASE V.—Young man has suffered from colic for about two years. Colic begins at the navel and passes down into the legs. Pains make him feel as if he was cut in two. He bends forward, pressing on his abdomen with his hands for relief. The pains come on about every three days. Took *one* dose of Colocynth 200. The pains, after taking the medicine, were very severe. After the attack passed off, took another dose. More than a year has passed by and he has had no return of the pains.

CASE VI.—Miss A. B. has toothache, right side, upper and lower back teeth. Pulsating pain going up into the head; worse when lying down quiet. Face red and swollen; painful when pressed with the hand. Olfaction of Bell. 30 removed the pain in ten minutes.

